# Simulation Analysis of Multi-Physical Field Coupling and Parameter Optimization of ECM Miniature Bearing Outer Ring Based on the Gas-Liquid Two-Phase Turbulent Flow Model

**DOI:** 10.3390/mi13060902

**Published:** 2022-06-07

**Authors:** Zhaolong Li, Wangwang Li, Bingren Cao

**Affiliations:** 1 Key Laboratory of Advanced Manufacturing Intelligent Technology of Ministry of Education, Harbin University of Science and Technology, Harbin 150080, China; 2 School of Mechanical and Power Engineering, Harbin University of Science and Technology, Harbin 150080, China; liwangwang19970214@163.com (W.L.); 1920110144@stu.hrbust.edu.cn (B.C.)

**Keywords:** ECM, gas–liquid two-phase turbulence model, miniature bearing outer ring, machining accuracy, central composite design

## Abstract

Electrochemical machining (ECM) is an essential method for machining miniature bearing outer rings on the high-temperature-resistant nickel-based alloy GH4169. However, the influence of electrolyte temperature distribution and bubble rate distribution on electrolyte conductivity in the ECM area could not be fully considered, resulting in the simulation model not being able to accurately predict the machining accuracy of the outer ring of the miniature bearing, making it challenging to model and predict the optimal process parameters. In this paper, a multiphysics field coupled simulation model of electric, flow, and temperature fields during the ECM of the miniature bearing outer ring is established based on the gas–liquid two-phase turbulent flow model. The simulation analyzed the distribution of electrolyte temperature, bubble rate, flow rate, and current density in the machining area, and the profile change of the outer ring of the miniature bearing during the machining process. The analysis of variance and significance of machining voltage, electrolyte concentration, electrolyte inlet flow rate, and interaction on the mean error of the ECM miniature bearing outer rings was derived from the central composite design. The regression equation between the average error and the process parameters was established, and the optimal combination of process parameters for the average error was predicted, i.e., the minimum value of 0.014 mm could be achieved under the conditions of a machining voltage of 16.20 V, an electrolyte concentration of 9.29%, and an electrolyte inlet flow rate of 11.84 m/s. This is important to improve the machining accuracy of the outer ring of the ECM miniature bearing.

## 1. Introduction

With the development of aerospace and automotive manufacturing and machinery production miniaturization, precision and complex internal characteristics of the small hole structure are more and more widely used, such as small holes in the reaming form of it can be applied to the outer ring of the miniature bearing [1]. GH4169 is a high-temperature-resistant nickel-based alloy material widely used to manufacture miniature bearing parts [2]. However, due to the high hardness of the alloy in the use of conventional machining methods challenging to the process, poor machining accuracy, and surface quality, the service life of miniature bearings is not long [3]. Electric discharge machining (EDM) and laser beam machining (LBM) are both thermal processes with high machining efficiency. The disadvantages are that they produce recast layers, heat-affected areas, and tensile residual stresses that also reduce the service life of micro bearings [4,5,6,7]. Electrochemical machining (ECM) is based on the principle of anodic dissolution for metal removal, independent of the hardness of the workpiece. It has become one of the main machining techniques for machining miniature bearing outer rings on the high-temperature-resistant nickel-based alloy GH4169 because of its advantage of no thermal damage [8,9,10]. There are still some problems to be solved. For example, the influence of electrolyte temperature distribution and bubble rate distribution on electrolyte conductivity in the ECM area cannot be fully considered, resulting in the simulation model not being able to accurately predict the machining accuracy of the outer ring of the miniature bearing. Therefore, it is difficult to model and predict the optimal process parameters.

In Deconck et al. [11,12,13], a simulation model for calculating the temperature distribution was developed, and it was pointed out that the electrolyte temperature distribution has an important influence on the machining accuracy. However, the model ignores the effect of electrolyte bubble rate distribution on machining accuracy and uses the laminar flow N–S model, which reduces the heat transfer effect. In Fang et al. [14], a multiphysics field coupled simulation model was proposed to predict the electrolytic machining accuracy. However, they treated the electrolyte as a single-phase flow and neglected the effect of electrolyte bubble rate distribution on machining accuracy. In Gomez-Gallegos et al. [15], a 3D coupled multiphysics field finite element model was developed to predict ECM accuracy. In Li et al. [16], a coupled model of the magnetic field, electric field, and electrolyte flow in ECM was developed to predict the accuracy of ECM. However, both of these researchers neglected the influence of the bubble rate distribution of the electrolyte in the machining area on the ECM accuracy, resulting in the simulation model’s inaccurate prediction of the ECM accuracy. Kozak et al. [17] and Mayank et al. [18] pointed out that the ECM accuracy is also affected by the bubble rate distribution of the electrolyte in the machining area. Some researchers have conducted studies on the bubble rate distribution of electrolytes in the machining area. In Shimasaki et al. [19] and Zhang et al. [20], the effect of bubbles generated by the electrolyte in the machining area on the accuracy of the ECM is observed using transparent electrodes. In Chang et al. [21], a two-dimensional two-phase laminar flow quasi steady-state flow model is proposed to predict the accuracy of the ECM. In Klocke et al. [22], the flow field in the machining area is assumed to be a gas–liquid two-phase flow. The bubble rate distribution in the electrolyte is approximated using the laminar bubble flow model. The use of the laminar N–S model reduces the heat transfer effect, making the prediction accuracy of the simulation model low. In Chen et al. [23] and Zhou et al. [24], a multiphysics field coupled simulation model for the ECM of turbine blades was established based on the gas–liquid two-phase turbulent flow k-ε model, and the effects of electrolyte temperature distribution and bubble rate distribution on the accuracy of the ECM of turbine blades were fully considered. The results show that the simulation model is highly accurate in predicting the machining accuracy of turbine blades.

The complexity of the ECM makes it challenging to model and predict the optimal process parameters [25]. In addition, the random selection of process parameters or trial-and-error methods is very costly and time-consuming and does not yield the desired results. These problems can be solved by optimization techniques [26,27]. Jain et al. [28] used a genetic algorithm to optimize the ECM process parameters. Three process parameters, namely, tool cathode feed rate, electrolyte flow rate, and machining voltage were selected as input quantities and machining accuracy as output quantities, and the optimization results obtained showed significant improvement in machining accuracy. In Jegan et al. [29], the optimization of the ECM process parameters based on the particle swarm algorithm was investigated. Four process parameters, namely, machining current, machining voltage, electrolyte concentration, and tool cathode feed rate were selected as input quantities and material removal rate and surface roughness as output quantities, and the particle swarm algorithm was determined to be superior to the genetic algorithm in terms of computation time and statistical analysis. In Mehrvar et al. [30], based on the central composite design to optimize the ECM process parameters, four process parameters, namely, machining voltage, tool cathode feed rate, electrolyte flow rate, and electrolyte concentration were selected as input quantities, and the material removal rate and surface roughness were determined as output quantities. The results show that the proposed optimization method is effective and suitable.

In summary, the simulation model of ECM of the microbearing outer ring under the influence of electrolyte temperature distribution and bubble rate distribution is established based on the gas–liquid two-phase turbulent flow k-ε model to predict its machining accuracy. It is also essential to optimize the process parameters of the ECM of the miniature bearing outer ring through central composite design to improve its machining accuracy. In this paper, a multiphysics field coupled simulation model of electric, flow, and temperature fields during the ECM of the miniature bearing outer ring is established based on the gas–liquid two-phase turbulent flow model. The influence of electrolyte temperature distribution and bubble rate distribution on the accuracy of the ECM is fully considered. The process of the ECM of miniature bearing outer rings was investigated using a central composite design, and a regression equation between the mean error and the process parameters was established. The optimal combination of process parameters for the mean error was predicted. 

## 2. Simulation Model of ECM of the Miniature Bearing Outer Ring

### 2.1. Geometric Model

**Figure 1:**
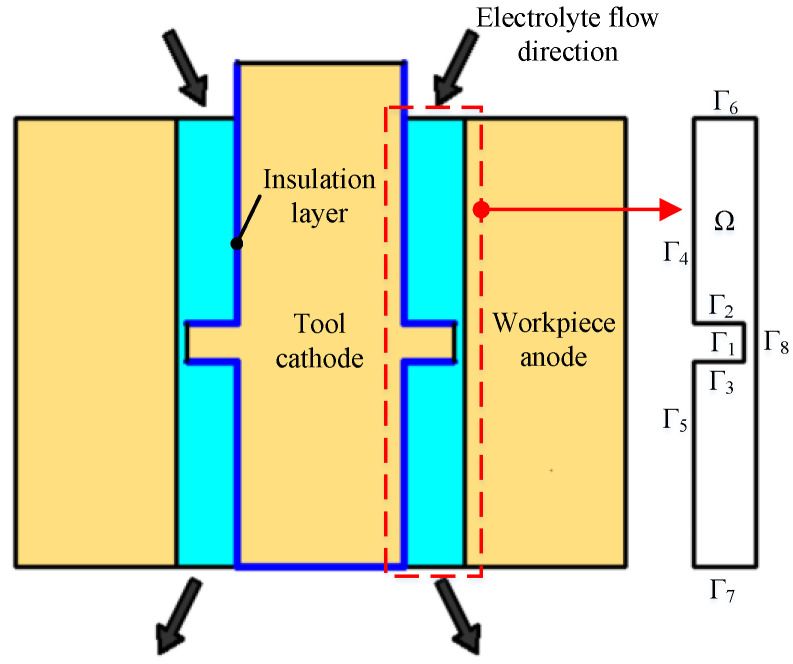
Geometric model of the machining area.

Since the entire ECM miniature bearing outer ring is modeled as an axisymmetric figure, it is assumed that the multiphysics field coupling is the same for each cross section. Therefore, the model is simplified in two dimensions for the convenience of analysis and calculation. The geometric model of the machining area is shown in Figure 1. The part inside the red dashed box in the figure is taken for analysis, and the shape of the entire machining area can be obtained by rotating it around the tool cathode axis for one week. Boundary Γ_1_ is the tool cathode without an insulating layer; boundaries Γ_2_, Γ_3_, Γ_4_, and Γ_5_ are the tool cathode with an insulating layer; boundary Γ_6_ is the electrolyte inlet; boundary Γ_7_ is the electrolyte outlet; boundary Γ_8_ is the workpiece anode, i.e., the outer ring of the miniature bearing, and Ω is the ECM area.

### 2.2. Mathematical Model

#### 2.2.1. Mathematical Model of Electric Field

According to Ohm’s law, the relationship between the current density in the machining area and the electric field strength and potential is:

(1) E=−∇φ

(2) i=σE=−σ∇φ

where *E* is the electric field strength; *φ* is the electrolyte potential; *i* is the current density; *σ* is the electrolyte conductivity.

In the actual ECM, the speed of the ECM is usually expressed in terms of the dissolution speed in the direction normal to the metal surface of the workpiece anode:

(3) $$v_{n}=\eta \omega i=-\eta \omega \sigma \nabla \varphi$$

where *v_n_* is the ECM speed; *η* is the ECM efficiency; *ω* is the volumetric galvanic equivalent of the workpiece anode.

#### 2.2.2. Mathematical Model of the Flow Field

The gas phase and solid phase products generated during the electrolysis process form a three-phase flow of gas, liquid, and solid in the machining area. In contrast, the volume ratio of the solid phase electrolysis products is minimal and has minimal effect on the electrolyte conductivity, so the flow field in the machining area can be simplified to a gas–liquid two-phase flow.

The gas–liquid two-phase flow in the ECM satisfies the conservation of mass:

(4) $$\frac{\partial }{\partial t}\left(\beta_{l}\rho_{l}+\beta_{g}\rho_{g}\right)+\nabla \cdot \left(\beta_{l}\rho_{l}u_{l}+\beta_{g}\rho_{g}u_{g}\right)=0$$

(5) $$\frac{\partial \beta_{g}\rho_{g}}{\partial t}+\nabla \cdot (\beta_{g}\rho_{g}u_{g})=m_{lg}$$

(6) $$\frac{\partial \beta_{l}\rho_{l}}{\partial t}+\nabla \cdot (\beta_{l}\rho_{l}u_{l})=-m_{lg}$$

(7) $$\beta_{l}+\beta_{g}=1$$

where *β_g_* is the proportion of gas in the total volume of the two-phase flow; *β_l_* is the proportion of liquid in the total volume of the two-phase flow; *ρ_g_* and *ρ_l_* are gas density and liquid density; *u_g_* and *u_l_* are the velocity of the gas phase and liquid phase; *m_lg_* is the mass transfer rate of the liquid phase into the gas phase.

The gas–liquid two-phase flow satisfies the conservation of momentum:

(8) $$\frac{\partial }{\partial t}\left(\beta_{g}\rho_{g}u_{g}\right)+\nabla \cdot (\beta_{g}\rho_{g}u_{g}u_{g})=-\beta_{g}\nabla p+\nabla \cdot \tau_{g}+\beta_{g}\rho_{g}g+F_{m}$$

(9) $$\frac{\partial }{\partial t}\left(\beta_{l}\rho_{l}u_{l}\right)+\nabla \cdot \left(\beta_{l}\rho_{l}u_{l}u_{l}\right)=-\beta_{l}\nabla p+\nabla \cdot \tau_{l}+\beta_{l}\rho_{l}g-F_{m}$$

where *p* is the electrolyte pressure; *τ_g_* and *τ_l_* are gas and liquid viscous stress tensors; *F_m_* is the interphase force.

Assuming that hydrogen is produced only on the surface of the tool cathode in the ECM and that the pressure and temperature distributions of the gas and liquid phases are the same, Faraday’s law states that:

(10) $$m_{H}=k_{H}It=k_{H}iSt$$

where *m_H_* is the mass of hydrogen produced; *k_H_* is the electrochemical equivalent of the hydrogen mass; *I* is the current; *i* is the current density; *S* is the area of the tool cathode.

The mass transfer rate *m_lg_* for the transformation of liquid phase into gas phase is the mass flux of hydrogen gas produced on the cathode per unit width of the tool, which combined with Equation (10) can be obtained after finishing:

(11) $$Lm_{lg}=ik_{H}$$

where *L* is the width of the tool cathode unit.

The density of hydrogen is calculated from the ideal gas equation of state as:

(12) $$\rho_{g}=\frac{p}{RT}$$

where *R* is the gas constant; *T* is the electrolyte temperature.

The electrolyte in the ECM region is in a turbulent state, and considering the effect of bubbles, the RANS *k*-*ε* turbulence model is used in this paper. The electrode surface near the wall is solved by the wall function as follows:

(13) $$\frac{\partial k}{\partial t}+\nabla \cdot \left[ku-\left(\mu +\frac{\mu_{T}}{\sigma_{k}}\right)\nabla k\right]=P_{k}+S_{k}-\varepsilon$$

(14) $$\frac{\partial \varepsilon }{\partial t}+\nabla \cdot \left[\varepsilon u-\left(\mu +\frac{\mu_{T}}{\sigma_{\varepsilon }}\right)\nabla \varepsilon \right]=\frac{\varepsilon }{k}\left(C_{1}P_{k}+C_{\varepsilon }S_{k}-C_{2}\varepsilon \right)$$

(15) $$P_{k}=\frac{\mu_{T}}{2}{\left|\nabla u+{\left(\nabla u\right)}^{T}\right|}^{2}$$

(16) $$S_{k}=-\beta C_{k}{\left|\nabla p\right|}^{2}$$

where *k* is the turbulent kinetic energy; *ε* is the turbulent dissipation rate; *u* is the electrolyte flow rate; *μ* is the electrolyte dynamic viscosity; *μ_T_* is the turbulent viscosity coefficient; *C*_1_, *C*_2_, *C_k_*, *C_ε_*, *σ_k_*, and *σ_ε_* are the model constants.

#### 2.2.3. Mathematical Model of the Temperature Field

During ECM, fluid heat transfer occurs in the machining area. The heat generated in the machining area is carried away by the thermal convection of the electrolyte in the turbulent state. According to the law of energy conservation, the expression of the convective heat transfer equation in the machining area is:

(17) $$\rho C_{p}\frac{\partial T}{\partial t}+\rho C_{p}u\cdot \nabla T=\nabla \left(\lambda \nabla T\right)+Q$$

(18) $$Q_{}=i\nabla \varphi$$

where *ρ* is the density of the electrolyte; *C_p_* is the specific heat capacity of the electrolyte; *λ* is the thermal conductivity of the electrolyte; *Q* is the heat generated in the ECM.

#### 2.2.4. Multiphysics Field Coupling Model

The conductivity of the electrolyte in the actual ECM as affected by temperature and bubble rate can be expressed as:

(19) $$\sigma =\sigma_{0}{\left(1-\beta \right)}^{m}\left[1+\gamma \left(T-T_{0}\right)\right]$$

where *σ_0_* is the initial conductivity of the electrolyte; *β* is the bubble rate; *m* is the bubble rate influence index; *γ* is the temperature correlation coefficient; *T*_0_ is the initial temperature of the electrolyte.

Substituting Equation (19) into Equation (3), the coupling equations for the electric field, flow field, temperature field, and ECM speed are obtained:

(20) $$v_{n}=-\eta \omega \sigma_{0}{\left(1-\beta \right)}^{m}\left[1+\gamma \left(T-T_{0}\right)\right]\nabla \varphi$$

### 2.3. 2D COMSOL Multiphysics Field Coupling Simulation Model

**Figure 2:**
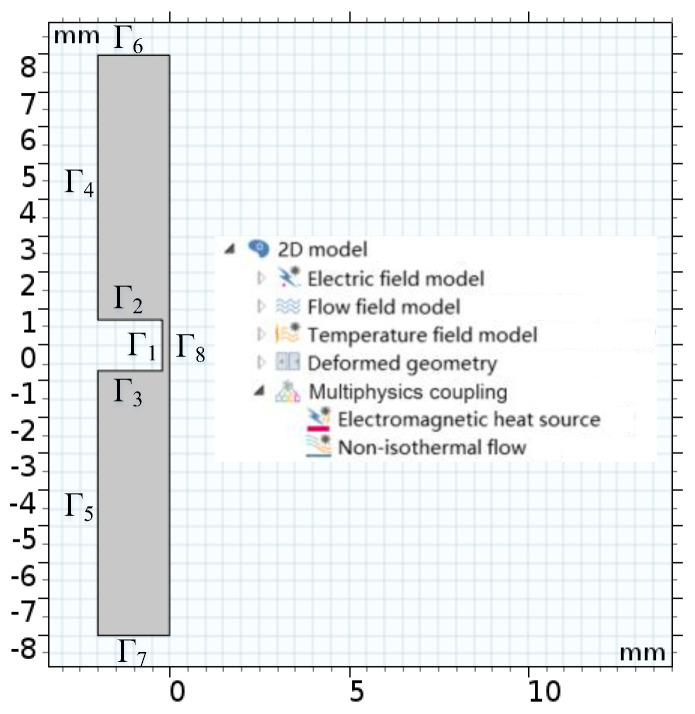
Simulation model and simulation module.

As shown in Figure 2, the electric field, flow field, temperature field, and deformation geometry modules on the software were selected for multiphysics field coupling simulation. The coupling method is that the electric and temperature field modules were coupled through an electromagnetic heat source module, and the flow and temperature field modules were coupled through a non-isothermal flow module. The actual ECM of the outer ring of the miniature bearing can be reproduced to the maximum extent.

#### 2.3.1. Boundary Condition Setting

The boundary conditions of the electric field module were set as follows: the boundary Γ_8_ was connected to the voltage U; the boundary Γ_1_ was grounded, and the boundaries Γ_2_, Γ_3_, Γ_4_, Γ_5_, Γ_6_, and Γ_7_ were electrically insulated. The boundary conditions of the flow field module were set as follows: boundary Γ_1_ gas mass flux, electrolyte wall function; boundary Γ_6_ electrolyte normal inflow velocity *u*_0_, no gas flux; boundary Γ_7_ electrolyte and gas outlet; and boundaries Γ_2_, Γ_3_, Γ_4_, Γ_5_, and Γ_8_ no gas flux, electrolyte wall function. The temperature module boundary conditions were set as follows: boundaries Γ_1_, Γ_2_, Γ_3_, Γ_4_, Γ_5_, and Γ_8_ thermal insulation; boundary Γ_6_ electrolyte initial temperature *T*_0_; boundary Γ_7_ electrolyte outflow. The boundary conditions of the deformation geometry module were set as follows: boundary Γ_8_ normal mesh moving speed, i.e., electrochemical processing speed *v_n_*.

#### 2.3.2. Material Parameter Setting of the Simulation Model

The workpiece anode material of the simulation model was GH4169 high-temperature-resistant nickel-based alloy; the tool cathode material was titanium alloy electrode (insulating film covered with PTFE), and the electrolyte was NaNO_3_ solution of a given concentration. The material parameters of the specific simulation model are shown in Table 1.

## 3. Simulation Analysis of ECM of the Miniature Bearing Outer Ring

The simulation conditions were as follows: machining voltage was 18 V; the electrolyte was NaNO_3_ solution with 12% concentration, and the electrolyte inlet flow rate was 9 m/s.

### 3.1. Electrolyte Temperature Distribution in the Machining Area

**Figure 3:**
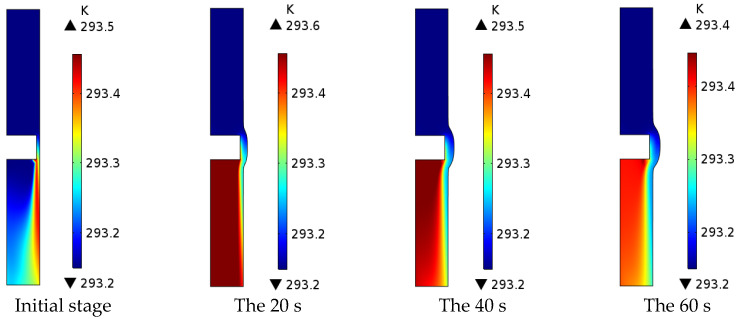
Electrolyte temperature distribution in the machining area.

As shown in Figure 3, the maximum electrolyte temperature in the machining area is 293.6 K, which is 0.45 K higher than the initial electrolyte temperature of 293.15 K. The maximum temperature of the electrolyte in the machining area from the initial stage of machining to the machining time of 20 s increased by 0.1 K, and the area of the thermally affected area was about 45% of the machining area. This is because more Joule heat is generated at this stage by higher current density, and the machining gap is small, resulting in poor electrolyte circulation and the accumulation of Joule heat in the direction of the electrolyte flow, causing the electrolyte temperature to increase and the area of the heat-affected area to expand. From the machining time of 20 s to the machining time of 60 s, the maximum temperature of the electrolyte in the machining area is reduced by 0.2 K, and the area of the heat-affected area is about 35% of the machining area. This is because the current density at this stage is low; the Joule heat generated is low, and the machining gap is large. The electrolyte flow is smooth, so that the electrolysis temperature is lower, and the area affected by heat is reduced.

### 3.2. Distribution of Electrolyte Bubble Rate in the Machining Area

**Figure 4:**
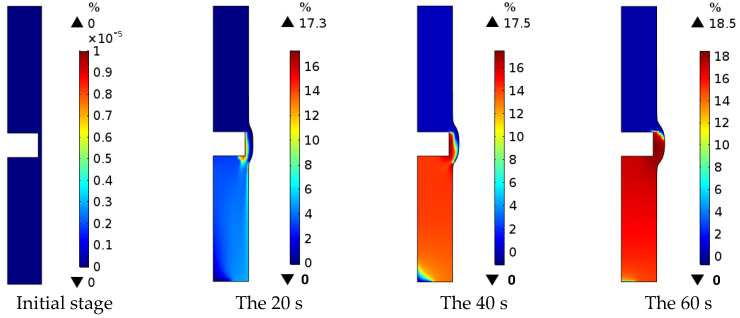
Distribution of electrolyte bubble rate in the machining area.

As shown in Figure 4, the bubble rate of electrolytes in the machining area gradually increases as the ECM proceeds. The highest bubble rate of electrolytes in the machining area reaches 18.5% at 60 s of machining time. The highest bubble rate of electrolytes in the machining area from the initial machining stage to 20 s of machining time reached 17.3%, and the electrolyte bubbles were distributed around the boundary Γ_1_ with a minimal area. This is because the high flow rate of the electrolyte at this stage can carry away the hydrogen produced at the boundary Γ_1_ in time. The highest bubble rate of electrolytes in the machining area increased by 1.2% from 20 s of machining time to 60 s of machining time, and the area of electrolyte bubble distribution was about 55% of the machining area. It is because the low flow rate of electrolyte at this stage is not able to take away the hydrogen produced by the boundary Γ_1_ in time, so the hydrogen accumulates along the electrolyte flow direction, causing the electrolyte bubble distribution area to expand.

### 3.3. Electrolyte Flow Rate Distribution in the Machining Area

**Figure 5:**
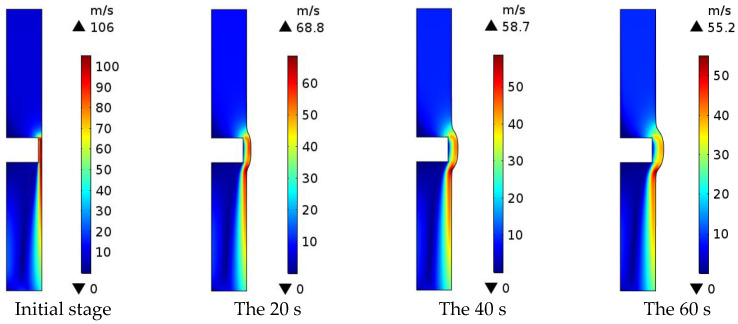
Distribution of electrolyte flow rate in the machining area.

As shown in Figure 5, the electrolyte flow rate in the machining area at the initial stage is high, up to 106 m/s. As the ECM progresses, the electrolyte flow rate gradually decreases, and the highest electrolyte flow rate in the machining area is 55.2 m/s when the machining time is 60 s. The electrolyte flow rate decreases as the machining gap increases, while the electrolyte flow rate remains constant.

### 3.4. Electrolyte Current Density Distribution and Workpiece Anode Profile Changes in the Machining Area

**Figure 6:**
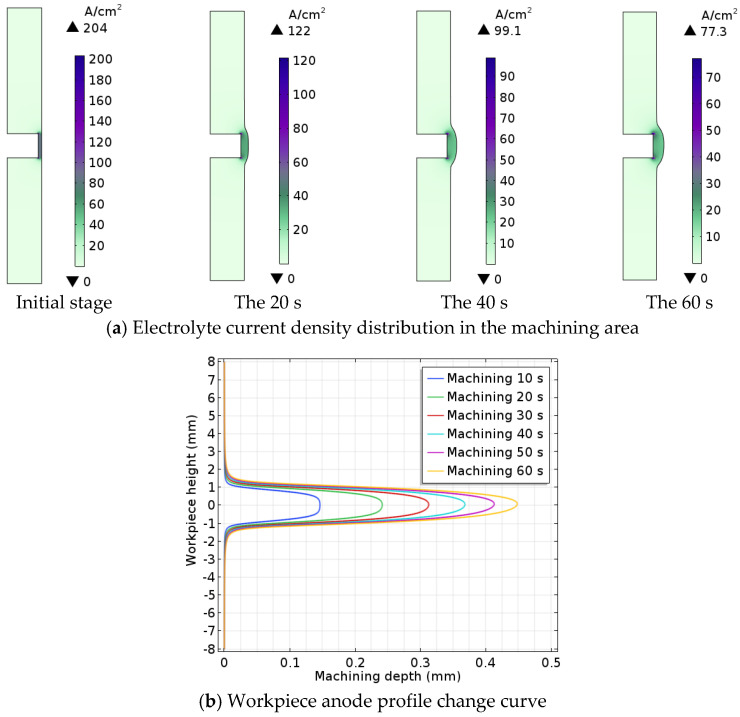
Electrolyte current density distribution in the machining area and workpiece anode profile change curve.

As shown in Figure 6a, the distance between the tool cathode and the workpiece anode gradually becomes larger. The electrolyte current density in the machining gap gradually decreases as the workpiece anode is dissolved during ECM. As shown in Figure 6b, the machining depth and height of the workpiece anode increase progressively as the ECM progresses, while the machining volume simultaneously tends to decrease. The main reason is that as the current density decreases during ECM, the speed of ECM becomes slower, and the machining volume declines simultaneously.

## 4. Process study of ECM of the Miniature Bearing Outer Ring

### 4.1. Process Evaluation Index for ECM of the Miniature Bearing Outer Ring

**Figure 7:**
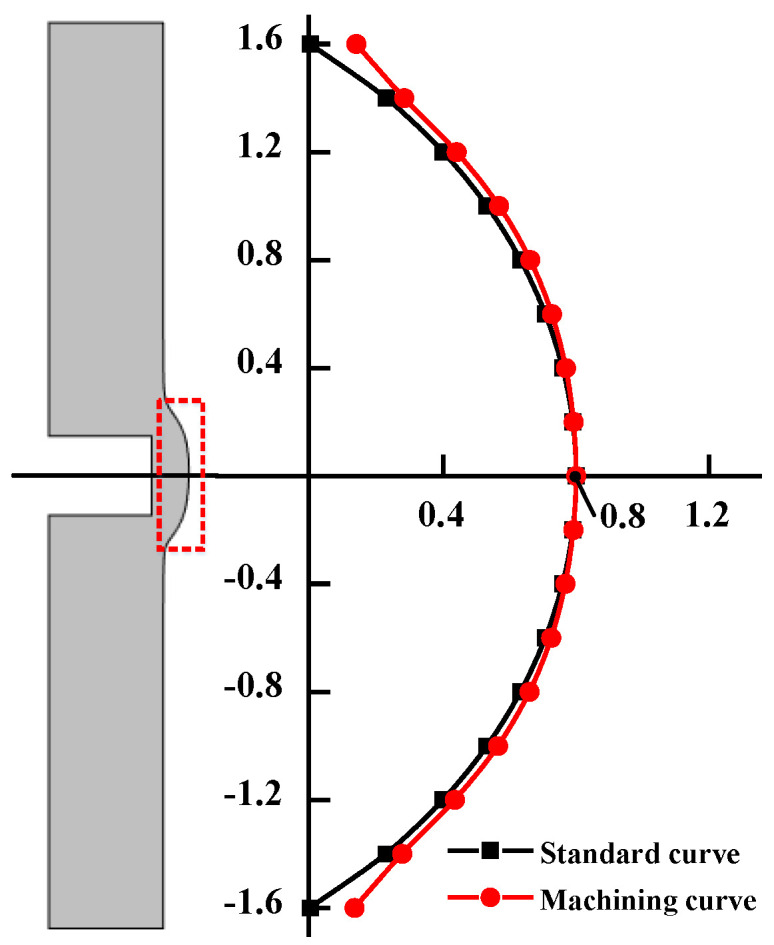
Standard curve and machining curve.

As shown in Figure 7, it is assumed that the equation of the standard curve of the outer ring section of the miniature bearing is: *x*^2^ + (*y* − 1.2)^2^ = 4, among them: 0 ≤ *x* ≤ 0.8. The cross-sectional curve of the ECM miniature bearing outer ring does not precisely coincide with the standard curve, and there is a specific error, so the average error of the process evaluation index of the ECM miniature bearing outer ring is constructed:

(21) $$\delta =\frac{1}{n}\sum_{1}^{n}|x_{b}-x_{a}|$$

where *δ* is the average error; *x_a_* is the horizontal coordinate of the standard curve; *x_b_* is the horizontal coordinate of the machining curve; *n* is the number of points taken uniformly along the vertical coordinate.

### 4.2. ECM Miniature Bearing Outer Ring Center Composite Design Solution

#### 4.2.1. Design Solutions and Simulation Results

In this design scheme, three process parameters, namely, machining voltage, electrolyte concentration, and electrolyte inlet flow rate were selected as input quantities. One process evaluation index, namely, average error was chosen as the output quantity. The process parameters were coded with −1, 0 with 1 representing the different level values of each process parameter, where “0” represents the center point of the level value; “−1” represents the low-level value; “1” represents the high-level value. The actual and coded values of the central composite design scheme are shown in Table 2. The center composite design scheme and simulation results are shown in Table 3.

#### 4.2.2. Establishing the Regression Equation

According to the results given in Table 3, the regression equation between the mean error of the process evaluation index of ECM miniature bearing outer ring and the process parameters was established through multiple quadratic orthogonal regression analysis:

(22) $$y=\alpha_{0}+\sum_{i=1}^{3}\alpha_{i}A_{i}+\sum_{i=1}^{3}\alpha_{ii}A_{i}^{2}+\sum \sum_{i<j}\alpha_{ij}A_{i}A_{j}+\gamma$$

where *y* is the output of the model; *α_0_* is a constant; αi is the primary term regression coefficient; *α_ii_* is the quadratic term regression coefficient; *α_ij_* is the interaction term regression coefficient; *γ* is the error estimate; *A_i_* is the coded value of the process parameters.

#### 4.2.3. Analysis of Variance and Significance of Each Factor

The regression analysis of the mean error of the outer ring of the ECM miniature bearing yielded the variance and significance analysis as shown in Table 4.

As shown in Table 4, the F-value of the model is 149.77 with a *p*-value less than 0.0001; the miss drafting F-value is 3.06, and the miss drafting *p*-value is 0.1226, indicating that the model is highly significant. The miss drafting term is not significant, showing that the model is meaningful and plausible. Simultaneously, R^2^ = 0.9926, Adjusted R^2^ = 0.9860, and Predicted R^2^ = 0.9718. The three values are similar and less different from 1, indicating that the model fits relatively well throughout the regression region. Adequate precision = 40.3255 and much greater than 4 indicates the model is more realistic and reliable. The established regression model has a good response. However, factors C, AC, and BC are not significant, so by excluding these insignificant factors, the regression model can be optimized.

#### 4.2.4. Optimization of Regression Models

As shown in Table 4, the impact of factors C, AC, and BC on the mean error was not significant, so the regression model was optimized using a stepwise elimination of insignificant factors to rerun the regression analysis. The variance and significance analysis of the mean error of the regression model after optimization are shown in Table 5.

As shown in Table 5, the F-value of the model is 290.62 with a *p*-value less than 0.0001; the miss drafting F-value is 1.92, and the miss drafting *p*-value is 0.2441, indicating that the model is highly significant. The miss drafting term is not significant, showing that the model is meaningful and plausible. Simultaneously, R^2^ = 0.9926, Adjusted R^2^ = 0.9892, and Predicted R^2^ = 0.9813. The three values are similar and less different from 1, indicating that the model fits relatively well throughout the regression region. Adequate precision = 54.6205 and much greater than 4 indicates that the model is more realistic and reliable. The response of the established regression model is good. All the factors are significant at this time.

#### 4.2.5. The Regression Equation of the Mean Error with the Normal Probability Distribution of the Residuals

According to the variance and significance analysis of the mean error of the optimized regression model, the calculation of each coefficient of the mean error regression equation was carried out, and the regression equation of the optimized mean error was obtained as:

(23) $$\begin{array}{l} \delta =0.0182+0.0050U+0.0055C+0.0054U\cdot C+\\ 0.0017U^{2}+0.0030C^{2}-0.0014V^{2} \end{array}$$

**Figure 8:**
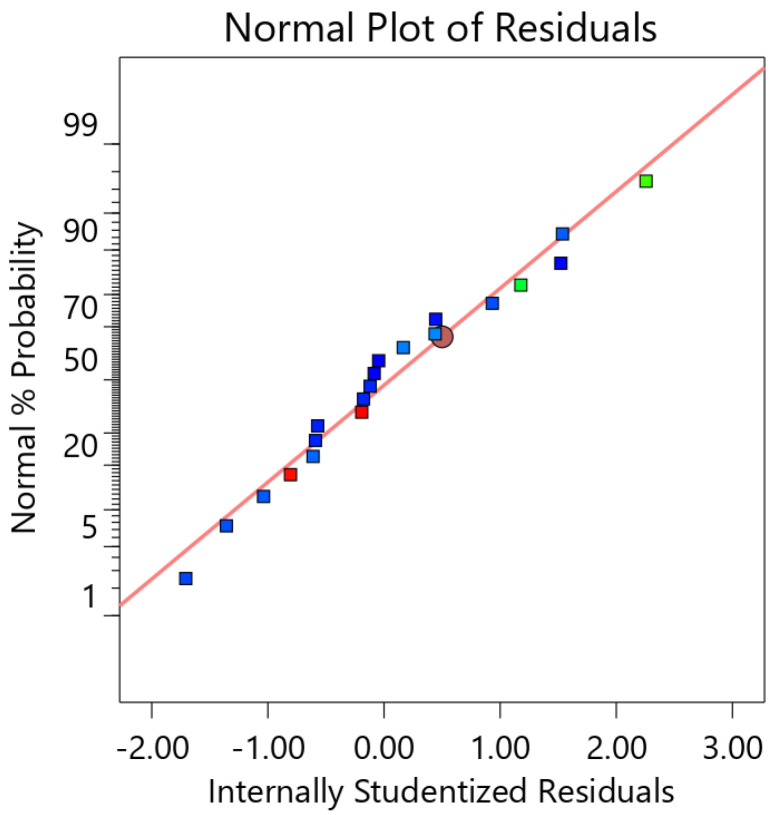
Normal probability distribution of residuals.

As shown in Figure 8, the residuals of each factor are distributed around a straight line. The residuals of each factor conform to a normal distribution, so the prediction of the mean error using Equation (23) is reliable.

#### 4.2.6. Response Surface Analysis

**Figure 9:**
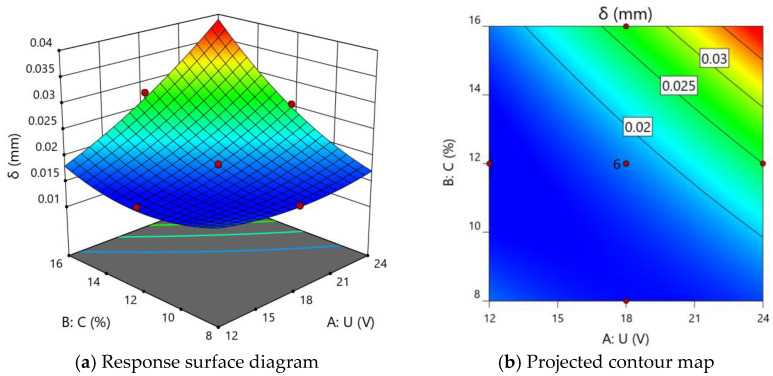
Effect of machining voltage and electrolyte concentration on the average error.

Since the effect of electrolyte inlet flow rate on the average error of the ECM of the miniature bearing outer ring is not significant, the inlet flow rate of electrolyte is taken as 9 m/s in the response surface analysis, and the effect of machining voltage and electrolyte concentration on the average error is shown in Figure 9.

As shown in Figure 9, the average error increases with the rise of processing voltage and electrolyte concentration. This is because both the increase of processing voltage and electrolyte concentration will increase the dissolution rate of the metal material on the anode surface of the workpiece, and when the processing depth is 0.8 mm, the radius of curvature of the cross-sectional curve of the outer ring of the miniature bearing produced by electrolysis increases, resulting in a rise in the average error between the standard curve and the standard curve.

#### 4.2.7. Prediction of the Best Combination of Process Parameters for the Average Error

**Figure 10:**
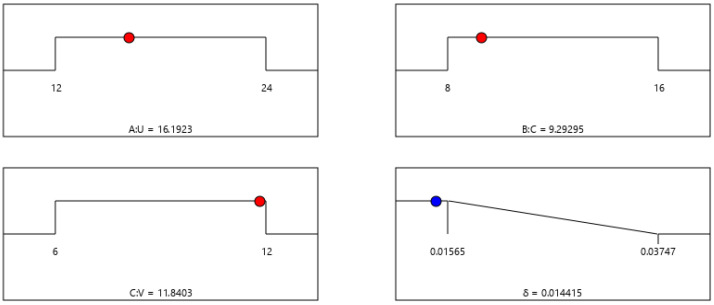
Optimal combination of process parameters for the average error.

The optimal combination of process parameters for the average error of the ECM miniature bearing outer ring was obtained using Design-Expert 12 software analysis as shown in Figure 10.

As shown in Figure 10, the average error of ECM of the miniature bearing outer ring can reach the minimum value of 0.014 mm under a machining voltage of 16.20 V, an electrolyte concentration of 9.29%, and an electrolyte inlet flow rate of 11.84 m/s.

## 5. Conclusions

In this paper, a multiphysics field coupled simulation model of electric, flow, and temperature fields during ECM of the miniature bearing outer ring is established based on the gas–liquid two-phase turbulent flow model. The process of ECM of miniature bearing outer rings is investigated using a central composite design. Then some conclusions were drawn from this study, which are summarized as follows:

1. In the ECM progresses, the depth and height of the workpiece anode gradually increase, while the machining volume simultaneously tends to decrease. The main reason is that the current density decreases as the electrolysis process progresses, resulting in a slower machining speed and a lower machining volume in the same amount of time.
2. The impact of machining voltage and electrolyte concentration on the average error was highly significant, while the impact of the electrolyte inlet flow rate on the average error was not significant.
3. When the electrolyte concentration is 12%, the electrolyte inlet flow rate is 9 m/s, and the machining voltage is increased from 12 V to 24 V, and the average error of the ECM of the miniature bearing outer ring is increased by 62.62%. When the machining voltage is 18 V, the electrolyte inlet flow rate is 9 m/s, and the electrolyte concentration is increased from 8% to 16%, and the average error of the ECM of the miniature bearing outer ring is increased by 74.22%. When the machining voltage is 18 V, the electrolyte concentration is 12%, and the electrolyte inlet flow rate is increased from 6 m/s to 12 m/s, and the average error of ECM of the miniature bearing outer ring is increased by 1.91%.
4. The average error of the ECM of the miniature bearing outer ring can reach the minimum value of 0.014 mm under a machining voltage of 16.20 V, an electrolyte concentration of 9.29%, and an electrolyte inlet flow rate of 11.84 m/s.

## Figures and Tables

**Table 1:** Material parameter settings of the simulation model.

Simulation Parameters	Numerical Value
Specific heat capacity of electrolyte (J/kg/K)	4200 1200
Electrolyte density (kg/m^3^)	
Thermal conductivity of electrolyte (W/m/K)	0.64
Electrolyte power viscosity (Pa·s)	1.01 × 10^−3^
The initial temperature of electrolyte (K)	293.15
Temperature correlation coefficient	0.025
Gas density (kg/m^3^)	8.99 × 10^−2^
Air bubble diameter (m)	1 × 10^−5^
Bubble rate impact index	1.5
GH4169 volumetric electrochemical equivalent (cm^3^/A/min)	0.00178

**Table 2:** Actual and coded values of the central composite design scheme.

Factors	Process Parameters	Codes		
		−1	0	1
A	Machining voltage U/V	12	18	24
B	Electrolyte concentration C/%	8	12	16
C	Electrolyte inlet flow rate V/m/s	6	9	12

**Table 3:** Center composite design scheme and simulation results.

No.	Factor A Machining Voltage U/V	Factor B Electrolyte Concentration C/%	Factor C Electrolyte Inlet Flow Rate V/m/s	Average Error δ/mm
1	12	8	6	0.01647
2	24	8	6	0.01565
3	12	16	6	0.01636
4	24	16	6	0.03747
5	12	8	12	0.0165
6	24	8	12	0.01567
7	12	16	12	0.01635
8	24	16	12	0.03715
9	12	12	9	0.01573
10	24	12	9	0.02558
11	18	8	9	0.01602
12	18	16	9	0.02791
13	18	12	6	0.01734
14	18	12	12	0.01767
15	18	12	9	0.01756
16	18	12	9	0.01835
17	18	12	9	0.01853
18	18	12	9	0.01784
19	18	12	9	0.01712
20	18	12	9	0.01735

**Table 4:** Variance and significance of the mean error.

Source Items	Square and	Degree of Freedom	Average Value	F-Value	*p*-Value	Significance
Model	0.0009	9	0.0001	149.77	<0.0001	Highly significant
A	0.0003	1	0.0003	396.82	<0.0001	Highly significant
B	0.0003	1	0.0003	476.83	<0.0001	Highly significant
C	2.500 × 10^−10^	1	2.500 × 10^−10^	0.0004	0.9845	Not significant
AB	0.0002	1	0.0002	374.83	<0.0001	Highly significant
AC	1.280 × 10^−8^	1	1.280 × 10^−8^	0.0202	0.8897	Not significant
BC	1.805 × 10^−8^	1	1.805 × 10^−8^	0.0285	0.8692	Not significant
A^2^	8.326 × 10^−6^	1	8.326 × 10^−6^	13.16	0.0046	Significant
B^2^	0	1	0	40.43	<0.0001	Highly significant
C^2^	5.467 × 10^−6^	1	5.467 × 10^−6^	8.64	0.0148	Significant
Residuals	6.328 × 10^−6^	10	6.328 × 10^−7^	——	——	——
Miss drafting	4.769 × 10^−6^	5	9.537 × 10^−7^	3.06	0.1226	Not significant
Pure error	1.559 × 10^−6^	5	3.118 × 10^−7^	——	——	——
Total	0.0009	19	——	——	——	——
R^2^ = 0.9926		Adjusted R^2^ = 0.9860		Predicted R^2^ = 0.9718		Adeq Precision = 40.3255

Where at *p* < 0.001, the factor is highly significant; at *p* < 0.05, the factor is significant; at *p* ≥ 0.05, the factor is not significant.

**Table 5:** Variance and significance of the mean error of the optimized regression model.

Source Items	Square and	Degree of Freedom	Average Value	F-Value	*p*-Value	Significance
Model	0.0009	6	0.0001	290.62	<0.0001	Highly significant
A	0.0003	1	0.0003	513.35	<0.0001	Highly significant
B	0.0003	1	0.0003	616.85	<0.0001	Highly significant
AB	0.0002	1	0.0002	484.90	<0.0001	Highly significant
A^2^	8.326 × 10^−6^	1	8.326 × 10^−6^	17.02	0.0012	Significant
B^2^	0	1	0	52.30	<0.0001	Highly significant
C^2^	5.467 × 10^−6^	1	5.467 × 10^−6^	11.18	0.0053	Significant
Residuals	6.359 × 10^−6^	13	4.891 × 10^−7^	——	——	——
Miss drafting	4.800 × 10^−6^	8	6.000 × 10^−7^	1.92	0.2441	Not significant
Pure error	1.559 × 10^−6^	5	3.118 × 10^−7^	——	——	——
Total	0.0009	19	——	——	——	——
R^2^ = 0.9926		Adjusted R^2^ = 0.9892		Predicted R^2^ = 0.9813		Adeq Precision = 54.6205

Where at *p* < 0.001, the factor is highly significant; at *p* < 0.05, the factor is significant; at Podel was optimized using a stepwise.

## Data Availability

Data are contained within the article.
